# Modern Brainstem MRI Techniques for the Diagnosis of Parkinson's Disease and Parkinsonisms

**DOI:** 10.3389/fneur.2020.00791

**Published:** 2020-08-04

**Authors:** Germain Arribarat, Amaury De Barros, Patrice Péran

**Affiliations:** ^1^ToNIC, Toulouse NeuroImaging Center, Université de Toulouse, Inserm, UPS, Toulouse, France; ^2^Centre de Recherche Cerveau et Cognition (CNRS, Cerco, UMR5549), UPS, Toulouse, France; ^3^Department of Anatomy, Toulouse Faculty of Medicine, Toulouse, France

**Keywords:** iron, neuromelanin (NM), multiple system atrophy (MSA), NODDI, diffusion kurtosis imaging (DKI), nigrosome

## Abstract

The brainstem is the earliest vulnerable structure in many neurodegenerative diseases like in Multiple System Atrophy (MSA) or Parkinson's disease (PD). Up-to-now, MRI studies have mainly focused on whole-brain data acquisition. Due to its spatial localization, size, and tissue characteristics, brainstem poses particular challenges for MRI. We provide a brief overview on recent advances in brainstem-related MRI markers in Parkinson's disease and Parkinsonism's. Several MRI techniques investigating brainstem, mainly the midbrain, showed to be able to discriminate PD patients from controls or to discriminate PD patients from atypical parkinsonism patients: iron-sensitive MRI, nigrosome imaging, neuromelanin-sensitive MRI, diffusion tensor imaging and advanced diffusion imaging. A standardized multimodal brainstem-dedicated MRI approach at high resolution able to quantify microstructural modification in brainstem nuclei would be a promising tool to detect early changes in parkinsonian syndromes.

## Introduction

In Parkinson's disease (PD), the first central neuropathological events occur in the brainstem and olfactory bulb. The dorsal motor nucleus of the vagal nerve in the medulla oblongata, which receives inputs from the neurenteric system, is classically considered to be the first nucleus involved before the ascending diffusion through other brainstem structures such as the serotoninergic raphe nuclei and noradrenergic coeruleus nuclei of the pons (1). Neurodegeneration then reaches the midbrain, and more particularly the dopaminergic substantia nigra pars compacta (SNc) and cholinergic pedunculopontine nuclei (PPN). The SNc plays a pivotal role, with classic PD motor symptoms occurring when 30% or more of its dopaminergic neurons have disappeared (2).

Before the appearance of these motor symptoms, the spread of alpha-synuclein across the brainstem following the Braak stages is responsible for many non-motor symptoms, such as cardiac autonomic dysfunction, rapid eye movement behavior disorder, apathy, asthenia, depression and dysexecutive syndrome, arising from alteration of the different nuclei and white-matter bundles of the brainstem (3, 4). The ensuing SNc degeneration caused by disruption of serotoninergic, extranigral dopaminergic, cholinergic, and noradrenergic pathways leads to the limbic and cognitive network dysfunction that has been amply described in numerous molecular imaging studies (5, 6). Recent advances in structural MRI techniques, such as water molecular diffusion based techniques and neuromelanin (NM) or iron-sensitive sequences, are resulting in the ever more precise characterization of brainstem damage in PD and atypical parkinsonism, including a more precise comprehension of brainstem networks involved in non-motor symptoms. When extrapyramidal motor symptoms are present, the challenge is to differentiate true PD from atypical parkinsonian syndromes, the most challenging of these being progressive supranuclear paralysis (PSP), Parkinsonian or cerebellar variant of multiple system atrophy (MSAp, MSAc), and dementia with Lewy bodies (DLB). *Red flags* for these syndromes in otherwise normal routine MRI sequences for PD often take the form of anatomical structure atrophy, reflecting the massive neuronal loss and gliosis that generally characterize the more advanced stages (for a review of classic MRI signs for differential diagnosis, see (7).

As these red flags are already familiar to clinicians, the present review focuses on modern structural MRI techniques for PD diagnosis and the differential diagnosis of atypical parkinsonism, considering structures involved in motor networks, but also those involved in non-motor networks.

## Nigrosome Imaging

Located in the midbrain, the susbtantia nigra (SN) is functionally and structurally divided into two parts: the SNc and the pars reticulata (SNr). The rostroventral GABAergic SNr projects toward the thalamus, and the dopaminergic SNc toward the striatum. Calbindin immunohistochemistry has allowed a labeled nigral matrix and five unlabeled clusters or nigrosomes to be identified within the SNc. These are compartments of dopaminergic neurons where degeneration is particularly marked in PD (8–10). Five nigrosomes measuring up to several millimeters long have been described (11), the largest of which (Nigrosome-1) is found in the dorsal region of the SNc.

The structure of Nigrosome-1 has been successfully delineated on 7T MRI using high-resolution susceptibility weighted imaging (SWI) (12, 13). On SWI, this nigrosome has a hypersignal in the axial section, in either linear or comma form. It is bordered anteriorly, laterally and medially by a low-intensity signal, giving it a *swallow tail* appearance. The absence of this sign is viewed as a reliable diagnostic criterion for PD (12, 14). Nigrosome-1 visualization at 7T has high diagnostic accuracy: sensitivity (100%), specificity (87–100%), positive predictive value (91–100%), and negative predictive value (100%) (15).

This structure has also been observed on SWI at 3T with reduced contrast (14, 16). The phase information, used here as a weighting mask, improves the visualization of the nigrosome's (16–18). This improvement stems from the difference in susceptibility between Nigrosome-1 and the surrounding nigral matrix in healthy individuals. As nigrosomes have a low iron concentration, they are visualized as a T2^*^ hypersignal, contrasting with the nigral matrix. Recently, a meta-analysis reporting different nigrosome imaging techniques confirmed that visual assessment of dorsolateral nigral hyperintensity provides excellent diagnostic accuracy for PD vs. controls (19).

The two purported mechanisms behind the disappearance of the swallow tail sign are an increase in iron and a decrease in NM. A decrease in NM can cause a decrease in iron retention capacity, and therefore an increase in the amount of free iron. In both cases, the presence of iron induces paramagnetic properties of the signal (20).

## Iron-Sensitive Imaging

During the last decade, many works, using different iron-sensitive MRI methods, confirmed the importance of nigral iron increase in PD patients compared to controls (21). Iron-sensitive MRI has several applications, especially in neurodegenerative disorders (22). Technically, iron content can be estimated in specific regions by measuring T2 and T2^*^ relaxation rates, using either magnitude (R2^*^) (23–25) or phase (quantitative susceptibility mapping, QSM) imaging (26). QSM method demonstrated to be the most sensitive quantitative technique for detecting a significant increase of iron for PD (27). QSM is able to detect nigral iron increase even in prodromal stage of PD such as idiopathic rapid eye movement sleep behavior disorder (28). It is important to note that iron-content in the substantia nigra do not differ between PD and multiple system atrophy (MSA) patients (i.e., patient with atypical parkinsonism), and between MSA variants (29). Accordingly, studies have shown that QSM more closely reflects levodopa dosage and disease severity (30, 31).

Concerning the differential diagnosis between PD and atypical parkinsonism's, QSM techniques allow for more sensitive measures in the nigral and extranigral regions. Mazzucchi et al. (32) found that the greatest diagnostic accuracy for PSP was for increased χ values in the RN, subthalamic nucleus (STN), and medial part of the SN, whereas for MSA, it was a significantly higher level of iron deposition in the putamen, reflecting the different patterns of pathological involvement that characterize these diseases (32).

## Neuromelanin-Sensitive Imaging

NM is a dark pigment composed of melanin, proteins, lipids and metal ions (33, 34). NM-containing neurons are particularly concentrated in the SNc and locus coeruleus (LC). This pigment is found in both the nigral matrix and the nigrosomes. The MR signal in NM-containing neurons is paramagnetic and therefore allows easy MR imaging where it is sensitive to this type of signal (35–37). T1-weighted NM-sensitive MRI (NM-MRI) produces hyperintense signals in regions containing NM. Indeed, the primary mechanism underlying contrast in NM-MRI appears to be the T1 reduction associated with melanin–iron complexes (38).

Studies have confirmed that patients with PD have a significantly reduced NM signal in the SN and LC (20, 37, 37, 39–41). These signal changes have been found to be correlated with the absorption values of the nigrostriatal dopamine transporter (42). In this same study, volumetric analysis of the NM-related signal also revealed a significant degree of atrophy. Measurement of NM-sensitive images has high diagnostic accuracy for PD. Several teams have recently evaluated a multimodal methodological approach, combining diffusion and NM-sensitive MRI. Pyatigorskaya et al. (43) concluded that volume delineation by the NM signal, combined with fractional anisotropy, has excellent diagnostic accuracy for PD. More specifically, NM signal intensities can potentially be located in the SNc (43). Another study found a lower NM signal in PD and MSAp groups than in an PSP group, with lower intensities in the LC in patients with PD. Sensitivity and specificity were 60 and 90% for PD vs. MSAp, 63–88% and 77–92% for PD vs. PSP, and 80 and 85% for MSAp vs. PSP (44).

## Diffusion Weighted Imaging

### Diffusion Tensor Imaging (DTI)

There are conflicting findings concerning the usefulness of SN DTI metrics in PD diagnosis, according to recent meta-analyses (45–47) and recent voxelwise analysis-based studies concerning the whole SN (48–50). Fractional Anisotropy (FA) in the posterior part seems to be the most discriminant feature (51, 52). With the ADC for the whole SN, Zhong et al. (53) found accuracy of 0.72, which is not sufficient in routine diagnosis. Interestingly, DTI metrics have also been used in a longitudinal approach, providing precious information on structural changes in the SN over the years (53–56).

In a longitudinal study applying a voxelwise approach, Pozorski et al. (50) found that patients with PD and controls differed on all DTI metrics in different brainstem regions (midbrain and pontine tegmentum, pontine crossing tract, periaqueductal gray matter). Furthermore, Mean diffusivity (MD) in the brainstem were negatively associated with disease duration. Pyatigorskaya et al. (57) correlated DTI metrics in the medulla oblongata with cardiac and respiratory variability in patients with PD compared with controls, reflecting their autonomic dysfunction (Stage 1) (57). Prange et al. (58) correlated apathy and depression with DTI measures and serotoninergic molecular imaging in the limbic system, and with the mode of anisotropy (sensitive to the orientation of crossing fibers) in the caudal midbrain, or more specifically the serotoninergic raphe nuclei (Stage 2) (58). Rapid eye movement behavior disorder has also been linked to DTI changes in the pons, SN and LC (59–61). Freezing of gait has been correlated with an FA decrease and MD increase in the PPN (62).

Diffusivity in the striatum, brainstem and cerebellum has been extensively studied (7, 63), but with rather mediocre results for the differential diagnosis of PD. FA or MD in the brainstem can be included in a multimodal approach with relatively good accuracy (49, 64). For example, the area under the curve (AUC) was above 0.95 for the diagnosis of PD vs. MSA in Péran et al. (49). Combining FA and MD in multiple ROIs including brainstem structures, Du et al. (51) were able to discriminate parkinsonian syndromes. With R2^*^, the AUC rose to 0.98–0.99. Pyatigorskaya et al. (43) recently found substantial differences in FA between patients with PSP and patients with PD or controls in the SN, LC, midbrain tegmentum, and pons. The highest AUC for PD vs. PSP was in the LC (0.94). In the same vein, Talai et al. (65) found AUCs of 0.95 and 0.97 based on DTI metrics in the midbrain for PD vs. PSP. DTI abnormalities in the midbrain of patients with PSP reflect microstructural changes that precede the macrostructural changes revealed by midbrain atrophy (65).

### Advanced Diffusion Approaches

Despite unique insights yielded by DTI metrics acquired in a single shell, microstructural changes are considered non-specific.

Recently, bi-tensor model have been used on diffusion imaging is to differentiate free water (FW) (cerebrospinal fluid or extracellular vasogenic edema) from other compartments, and either eliminate it from DTI measures or directly map it. Although it was initially developed with a single shell (66), double-shell acquisition could offer greater accuracy and stability (67). The most striking feature is the FW increase in the posterior part of the SN in early PD (68), MSA and PSP (69), as confirmed by (70). Findings of longitudinal FW changes over 4 years in the posterior SN of patients with early PD (71), followed by an FW increase over 3 years in the anterior SN of patients with advanced PD (72), emphasize the usefulness of FW imaging for PD diagnosis and follow-up. This posterior-anterior gradient of extracellular space expansion could reflect the neuroinflammation and cell loss revealed by histopathology. Increased FW in the posterior SN has been correlated with striatal dopaminergic denervation and reflects both motor and cognitive deficits (73). Planetta et al. (74) found an FW increase in the SN, as well as in the STN, red nucleus, PPN, cerebellum and basal ganglia, in patients with PSP and MSA, compared with patients with PD and controls. FW in the PPN and STN was discriminant for the diagnosis of MSA versus PSP (AUC = 0.97) (74).

Estimating kurtosis provides a better means of assessing diffusion heterogeneity in tissues. Diffusion kurtosis imaging (DKI) requires diffusion acquisition in two shells (often b = 1,000 and 2,000 s/mm2) (75). Elevated mean kurtosis in the SN has been found to be more sensitive than FA reduction in patients with PD versus controls (AUC > 0.95) and is correlated with motor scores (76).

White-matter tissue has been simplified in a model with intra-axonal, extra-axonal, and cerebrospinal fluid compartments. Neurite orientation dispersion and density imaging (NODDI) takes all three compartments into account. Three new metrics can be extracted from NODDI: isotropic volume fraction (Viso), reflecting extracellular space like FW; intracellular volume fraction (Vic); and orientation dispersion index (ODI), reflecting neurite integrity. An ODI increase in white matter reflects axonal loss or disorganization, while ODI and Vic decreases in gray-matter structures reflect dendritic thinning and density (77). Kamagata et al. (78) found that Vic in the contralateral SNc is useful for diagnosing patients with PD vs. controls (AUC = 0.91) and correlates with disease severity (78). NODDI was recently compared with FW for diagnosing PD, PSP and MSAp. For PD and MSAp, Viso in the posterior SN was increased (FW accumulation). In PSP, all three NODDI metrics were disturbed in the whole SN, STN, RN and PPN. Interestingly, the authors considered the NODDI metrics to be inferior to FW for PD vs. MSAp/PSP diagnosis, and found that FW based on a single shell was just as accurate and actually faster than multishell FW (79).

Restriction spectrum imaging (RSI) (80, 81) is a new diffusion model based on high angular resolution diffusion imaging (HARDI), acquired in multiple directions at different high b values. RSI highlights hindered and restricted diffusion in extracellular and intracellular compartments. With the development of several new metrics, RSI can now provide information about neurite density and orientation. Hope et al. (81) were the first to compare RSI with DTI in patients with PD. They defined a cellularity index and a neurite density index. In an ROI comprising the whole brainstem, they found significant differences in the cellularity index between patients with PD and controls (AUC = 0.69). RSI is a promising new, but time-consuming, method for vulnerable patients. Further research with more precise anatomical ROIs is needed.

DWI offers the possibility of visualizing white-matter tracts with a multitude of tractography techniques. HARDI-based tractography allows complex fiber tracts such as the nigrostriatal pathways (NSPs) to be reconstructed (82, 83). An FA decrease and an AD/RD increase have been found in the NSPs of patients with PD using deterministic tractography (84). Based on NODDI, Andica et al. (85) found that the contralateral distal (relative to the striatum) Vic of NSPs was significantly decreased in patients with PD compared with controls, reflecting the *dying back* of dopaminergic neurons (85). From our point of view, the deterministic tractography of NSPs described in these studies does not render the exact structural connectivity between the striatum, pallidum and SN revealed by a probabilistic approach (82) or by susceptibility imaging (86). The tract-based approach to PD diagnosis is promising but needs further clarification of mesencephalon/diencephalon structural connectivity.

The dentatorubrothalamic tract (DRTT) connects the cerebellum with the thalamus via the superior cerebellar peduncle and tegmental midbrain. An FA decrease and an MD increase have been found in the DRTT of patients with PSP, compared with controls and patients with PD or MSAp (64, 87). In patients with MSAc, an FA decrease and an MD increase in the pontine crossing tract and middle cerebellar peduncles have been found before the appearance of the *hot cross bun* sign (88, 89). Using diffusion kurtosis imaging, Ito et al. (90, 91) described a ratio of midbrain tegmentum diffusion to pontine crossing tract diffusion to distinguish between patients with PSP, MSA or PD and controls (90, 91). Interestingly, Juttukonda et al. (92) also found an FA decrease in the pontine crossing tract in patients with essential tremor vs. PD (92).

## Conclusion

**Figure 1:**
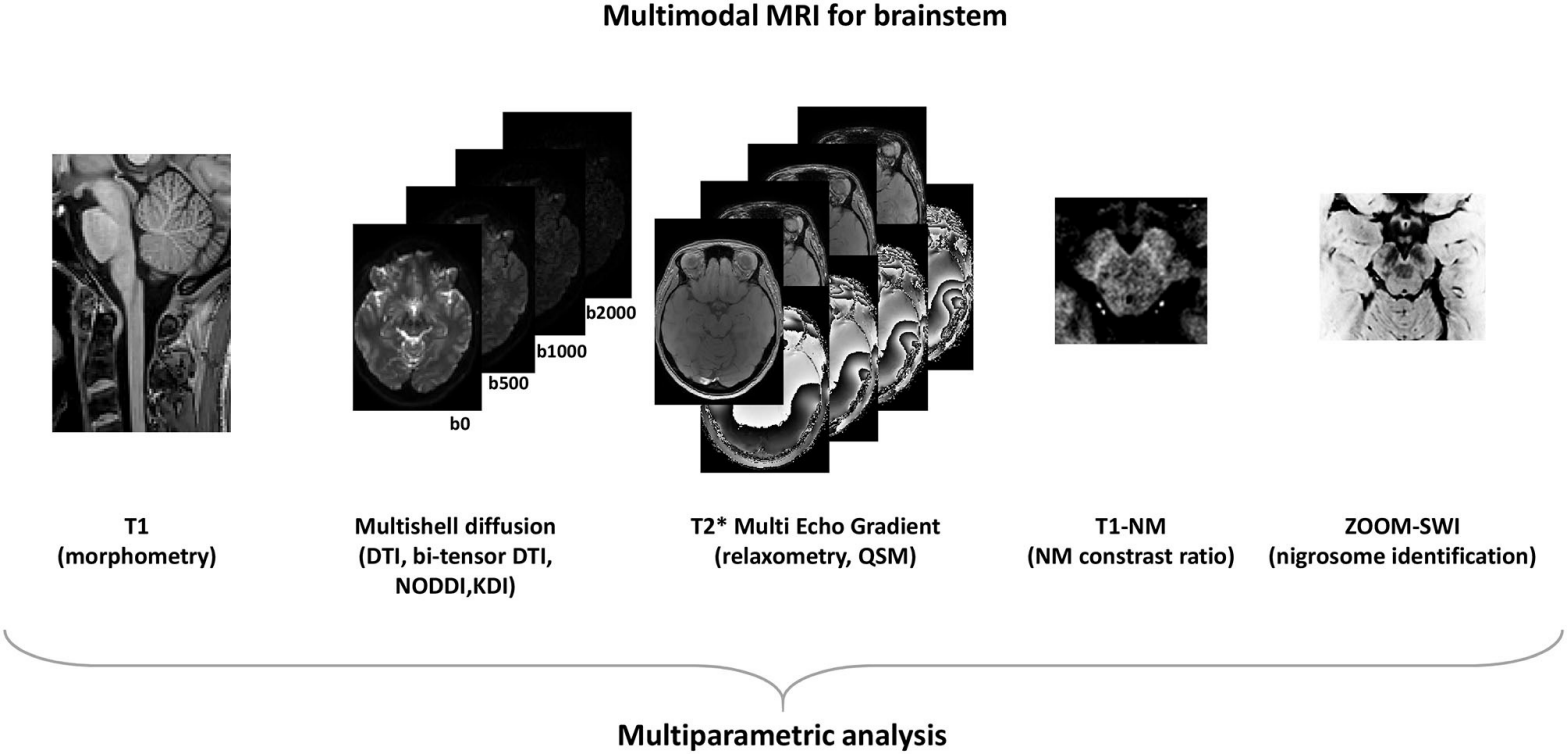
Modern MRI acquisitions to asses brainstem.

An important consideration is that MRI quantitative markers with good performances for diagnosis are not necessarily the best suited to monitor disease progression. More efforts need to be done in order to increase reliably and sensitivity of progression MRI markers of PD (93). A standardized multimodal brainstem-dedicated MRI approach at high spatial resolution (Figure 1) able to quantify microstructural modification in brainstem nuclei would be a promising tool to detect early changes in PD and parkinsonism and to follow disease progression.

## Author Contributions

AD, GA, and PP contributed conception and design of the review. AD and GA wrote the first draft of the manuscript. PP reviewed the first draft. All authors contributed to manuscript revision, read, and approved the submitted version.

## Conflict of Interest

The authors declare that the research was conducted in the absence of any commercial or financial relationships that could be construed as a potential conflict of interest.
